# Body and Pectoral Fin Kinematics During Routine Yaw Turning in Bonnethead Sharks (*Sphyrna tiburo*)

**DOI:** 10.1093/iob/obz014

**Published:** 2019-06-22

**Authors:** S L Hoffmann, M E Porter

**Affiliations:** Department of Biological Sciences, Florida Atlantic University, 777 Glades Road, Boca Raton, FL 33431, USA

## Abstract

Maneuvering is a crucial locomotor strategy among aquatic vertebrates, common in routine swimming, feeding, and escape responses. Combinations of whole body and fin movements generate an imbalance of forces resulting in deviation from an initial path. Sharks have elongate bodies that bend substantially and, in combination with pectoral fin rotation, play a role in yaw (horizontal) turning, but previous studies focus primarily on maximal turning performance rather than routine maneuvers. Routine maneuvering is largely understudied in fish swimming, despite observations that moderate maneuvering is much more common than the extreme behaviors commonly described in the literature. In this study, we target routine maneuvering in the bonnethead shark, *Sphyrna tiburo*. We use video reconstruction of moving morphology to describe three-dimensional pectoral fin rotation about three axes to compare to those previously described on yaw turning by the Pacific spiny dogfish. We quantify kinematic variables to understand the impacts of body and fin movements on routine turning performance. We also describe the anatomy of bonnethead pectoral fins and use muscle stimulation to confirm functional hypotheses about their role in actuating the fin. The turning performance metrics we describe for bonnethead sharks are comparable to other routine maneuvers described for the Pacific spiny dogfish and manta rays. These turns were substantially less agile and maneuverable than previously documented for other sharks, which we hypothesize results from the comparison of routine turning to maneuvering under stimulated conditions. We suggest that these results highlight the importance of considering routine maneuvering in future studies. Cinemática del Cuerpo y de las Aletas Pectorales Durante el giro en el eje Vertical en la Cabeza del Tiburón Pala (*Sphyrna tiburo*) (Body and Pectoral Fin Kinematics During Routine Yaw Turning in Bonnethead Sharks [*Sphyrna tiburo*])

## Synopsis

Maniobrar es una estrategia locomotora crucial entre los vertebrados acuáticos, la usan communmente al nadar, alimentarse y escapar. Las combinación de movimientos de todo el cuerpo y las aletas generan un desequilibrio de fuerzas que resulta en una desviación de una trayectoria inicial. Los tiburones tienen cuerpos alargados que se doblan sustancialmente y, en combinación con la rotación de la aleta pectoral, desempeñan un papel en el giro de horizontal de la cabez. Estudios anteriores se centraron principalmente en el rendimiento máximo de giro en lugar de las maniobras de rutina. Las maniobras de rutina son poco estudiadas en la natación de peces, a pesar de las observaciones de que las maniobras moderadas son mucho más comunes que las conductas extremas comúnmente descritas en la literatura. Utilizamos la reconstrucción con video de la morfología en movimiento para describir la rotación de la aleta pectoral tridimensional en tres ejes para compararla con los descritos anteriormente en un estudio sobre el giro de rutina realizado por el tiburón espinoso del Pacífico. Cuantificamos las variables cinemáticas para comprender los impactos de los movimientos del cuerpo y las aletas en el rendimiento de giro de rutina. También describimos la anatomía de las aletas pectorales tiburón cabeza de pala y utilizamos la estimulación muscular para confirmar las hipótesis funcionales sobre su papel en la actuación de la aleta. Las métricas de rendimiento de giro que describimos para los tiburones cabeza de pala son comparables a otras maniobras de rutina descritas para el perrito espinoso del Pacífico y las mantas rayas. Estos giros fueron sustancialmente menos ágiles y maniobrables de lo que se documentó anteriormente para otros tiburones, lo cual, según nuestra hipótesis, resulta de la comparación del giro rutinario a la maniobra en condiciones estimuladas. Sugerimos que estos resultados resaltan la importancia de considerar maniobras rutinarias en estudios futuros. Translated to Spanish by J. Heras (herasj01@gmail.com)

Körper- und Brustflossenkinematik Beim Routinemäßigen Gieren von Schaufelnasen-Hammerhaien (*Sphyrna tiburo*) (Body and Pectoral Fin Kinematics During Routine Yaw Turning in Bonnethead Sharks [*Sphyrna tiburo*]) Das Manövrieren ist eine entscheidende Bewegungsstrategie bei aquatischen Vertebraten, die beim routinemäßigen Schwimmen, Fressen und Fluchtverhalten häufig vorkommt. Kombinationen von Ganzkörper- und Flossenbewegungen erzeugen ein Kräfteungleichgewicht, das zu einer Abweichung von einem initialen Weg führt. Haie haben lang gestreckte Körper, die sich erheblich biegen und in Kombination mit der Drehung der Brustflosse eine Rolle beim Gieren (horizontales Drehen) spielen. Bisherige Studien konzentrieren sich jedoch eher auf maximale Drehleistung als auf Routinemanöver. Das routinemäßige Manövrieren wurde beim Schwimmen von Fischen wenig untersucht, obwohl beobachtet wurde, dass mäßiges Manövrieren weitaus häufiger vorkommt als die in der Literatur häufig beschriebenen extremen Verhaltensweisen. Wir verwenden die Video-Rekonstruktion von bewegter Morphologie (video reconstruction of moving morphology, um die dreidimensionale Drehung der Brustflosse um drei Achsen zu beschreiben und diese mit den bisher in einer Studie über routinemäßige Drehung bei einer Art von Dornhaien beschriebenen zu vergleichen. Wir quantifizieren kinematische Variablen, um die Auswirkungen von Körper- und Flossenbewegungen auf die routinemäßige Drehleistung zu verstehen. Wir beschreiben außerdem die Anatomie der Brustflossen der Schaufelnasen-Hammerhaie und verwenden Muskelstimulation, um funktionelle Hypothesen über ihre Rolle beim Auslösen der Flossenbewegung zu bestätigen. Die von uns beschriebenen Kennzahlen für die Drehleistung von Schaufelnasen-Hammerhaien sind vergleichbar mit anderen Routinemanövern, die für die erwähnte Art Dornhaie und Mantarochen beschrieben wurden. Diese Drehungen waren wesentlich weniger wendig und manövrierfähig als zuvor für andere Haie dokumentiert, was sich nach unserer Hypothese aus dem Vergleich von routinemäßigen Drehungen und dem Manövrieren unter stimulierten Bedingungen ergibt. Wir legen nahe, dass diese Ergebnisse die Bedeutung der Berücksichtigung von Routinemanövern in zukünftigen Studien unterstreichen. Translated to German by F. Klimm (frederike.klimm@biologie.uni-freiburg.de)

Cinemática do Corpo e da Nadadeira Peitoral Durante a Rotação de Rotina em Tubarões Cabeça-de-Boné (*Sphyrna tiburo*) (Body and Pectoral Fin Kinematics During Routine Yaw Turning in Bonnethead Sharks [*Sphyrna tiburo*]) A manobra é uma estratégia locomotora crucial entre os vertebrados aquáticos, comum na natação rotineira, na alimentação e na resposta à ameaças. Combinações de movimentos do corpo e das nadadeiras geram um desequilíbrio de forças resultando em desvio do caminho inicial. Os tubarões têm corpos alongados que se dobram substancialmente e, em combinação com a rotação da nadadeira peitoral, desempenham um papel importante no giro horizontal (guinada), porém, estudos anteriores focaram principalmente no desempenho de giro máximo em vez de manobras rotineiras. A manobra rotineira é pouco estudada na natação de peixes, apesar das observações de que manobras moderadas são muito mais comuns do que os comportamentos extremos comumente reportados em literatura. Usamos a reconstrução de vídeo de morfologia móvel para descrever a rotação tridimensional da nadadeira peitoral para comparar com aqueles previamente descritos em um estudo sobre rotação rotineira pelo tubarão galhudo do Pacífico. Quantificamos as variáveis cinemáticas para entender os impactos dos movimentos do corpo e das nadadeiras no desempenho rotineiro de giro. Descrevemos também a anatomia das nadadeiras peitorais do tubarão martelo e utilizamos a estimulação muscular para confirmar hipóteses funcionais sobre o seu papel na movimentação da nadadeira. As métricas de desempenho de giro que descrevemos para os tubarões martelo são comparáveis a outras manobras de rotina descritas para o tubarão galhudo do Pacífico e raias-manta. Esses turnos foram substancialmente menos ágeis e manobráveis do que o anteriormente documentado para outros tubarões, dados dos quais nós criamos a hipótese com os resultados da comparação da rotação de rotina para manobrar sob condições estimuladas. Sugerimos que esses resultados ressaltem a importância de considerar manobras rotineiras em estudos futuros. Translated to Portuguese by Diego Vaz (dbistonvaz@vims.edu)

## Introduction

The ability to maneuver is essential for prey location, predator avoidance, and routine navigation. Body caudal fin swimmers primarily maneuver by modulating movements of the body axis, which can result in banking (rolling of the body), or the classic c-start escape response (Domenici and Blake 1993; Webb 1997; Fish 2002; Domenici et al. 2004; Fish et al. 2003a, 2003b, 2006; Goldbogen et al. 2013; Segre et al. 2016). Another maneuvering strategy employs appendage movements to produce torques (Harris 1936; Walker 1971; Webb 1983; Wyneken 1997; Hove et al. 2001; Fish and Nicastro 2003; Lauder and Drucker 2004; Rivera et al. 2006). Sharks use both body bending and fin movements during yaw (horizontal) maneuvering, but studies of unsteady swimming in sharks primarily address maximal turning performance, prey seeking, or escape response behaviors (Kajiura et al. 2003; Domenici et al. 2004; Porter et al. 2009; Hoffmann et al. 2019). Despite those previous observations, fish infrequently perform rapid maneuvers in volitional swimming, instead relying on moderate turning behaviors for routine maneuvering (Webb 1991; Wu et al. 2007a). Here, we examine the role of whole-body axis kinematics and pectoral fin movements during routing yaw turning in bonnethead sharks (*Sphyrna tiburo*).

Sharks have moderately flexible, elongate bodies that bend substantially during maneuvering (Kajiura et al. 2003; Domenici et al. 2004; Porter et al. 2009). Increased velocity is usually correlated with increased frequency and amplitude of bending, which can also affect maneuvering (Webb and Keyes 1982; Domenici and Blake 1997). Lateral displacement of the body during undulation is related (in part) to vertebral and cross-sectional trunk morphology (Kajiura and Holland 2002; Kajiura et al. 2003; Domenici et al. 2004; Porter et al. 2009; Hoffmann et al. 2017). Among fishes, differences in body shape are well documented to affect maneuvering performance, which should not be generalized among morphologically distinct species (Webb 1984; Wardle et al. 1995; Sfakiotakis et al. 1999; Blake 2004; Webb and Weihs 2015).

Arguably considered a morphological extreme, hammerhead species (Family: Sphyrnidae) have dorso-ventrally compressed and laterally expanded heads termed cephalofoils (Nakaya 1995). Species in this family have increased body flexibility and maneuverability during turning in comparison to species that lack the cephalofoil (Kajiura and Holland 2002; Kajiura et al. 2003; Porter et al. 2009). Hammerhead species also have additional anterior axial body musculature that increases range of motion of the cephalofoil (Nakaya 1995). There are conflicting hypotheses about the potential advantage of the laterally expanded hammerhead cephalofoil in maneuvering: the wing like head shape may generate turning forces during banking thereby increasing maneuverability, but two hammerhead species are not observed to bank during prey searching, while a third species may bank during routine swimming (Thomson and Simanek 1977; Nakaya 1995; Kajiura and Holland 2002; Kajiura et al. 2003; Payne et al. 2016). Instead, it appears that during turning, the pectoral fin located on the inside of the body curvature (hereafter referred to as the inside fin) may be moved to create a pivot about which the body bends, creating a smaller turning radius (Kajiura et al. 2003). Based on the presence of increased anterior axial musculature in hammerhead species, we hypothesize that the bonnethead shark may also have increased muscular control over the pectoral fins to facilitate turning.

At least two shark species (the bonnethead and Pacific spiny dogfish) move their pectoral fins asynchronously during yaw maneuvering, and these fins are hypothesized to play a role in turning (Kajiura et al. 2003; Domenici et al. 2004; Hoffmann et al. 2019). During vertical maneuvering, pectoral fins generate thrust to reorient the body (Wilga and Lauder 2000, 2001), and asynchronous pectoral fin movement may create an imbalance of forces that increase maneuverability, similar to maneuvering mechanisms in other aquatic organisms (Gerstner 1999; Walker 2000; Drucker and Lauder 2001, 2004; Fish and Nicastro 2003; Fish et al. 2003a; Rivera et al. 2006; see Fish and Lauder [2017] for review). Further, pectoral fin actuation is under muscular control; thus, fin actuation may lead to a finer degree of control during maneuvering (Maia et al. 2012; Hoffmann et al. 2019). However, the combined role of the body, caudal fin, and pectoral fins in maneuvering largely remains unexplored for sharks.

The goals of this study were to describe the body and pectoral fin as they relate to turning performance of bonnethead sharks. We hypothesized that the bonnethead shark would protract, supinate, and depress the fin inside to body curvature, and that increasing fin rotation would correlate with turning performance metrics as previously described for the Pacific spiny dogfish (Hoffmann et al. 2019). Additionally, we documented whole body kinematics and investigated the combined effects of body bending, pectoral fin movement, swimming speed, and caudal fin movement on two metrics of turning performance. We also describe the anatomy of the bonnethead pectoral fin and used post mortem muscle stimulation to confirm hypotheses about the function of the pectoral fin musculature. We hypothesized that bonnethead sharks would have increased fin rotation compared with Pacific spiny dogfish based on previous studies suggesting they may rely solely on pectoral fin rotation for turning to avoid banking (Kajiura et al. 2003; Hoffmann et al. 2019).

## Materials and methods

Bonnethead sharks (*S.tiburo*, *n* = 4) were captured via gill net in Long Key, FL and transported to the Florida Atlantic University Marine Research Laboratory in Boca Raton, FL where they were cared for under FAU IACUC protocol A15-43. Individuals were all female and ranged in total length (TL) from 77.1 to 83.5 cm. Animals were housed in a 6-m diameter tank with 1.5 m water depth and flow-through seawater, and individuals were acclimated for minimum 7 days prior to filming trials.

### Marker placement

**Fig. 1:**
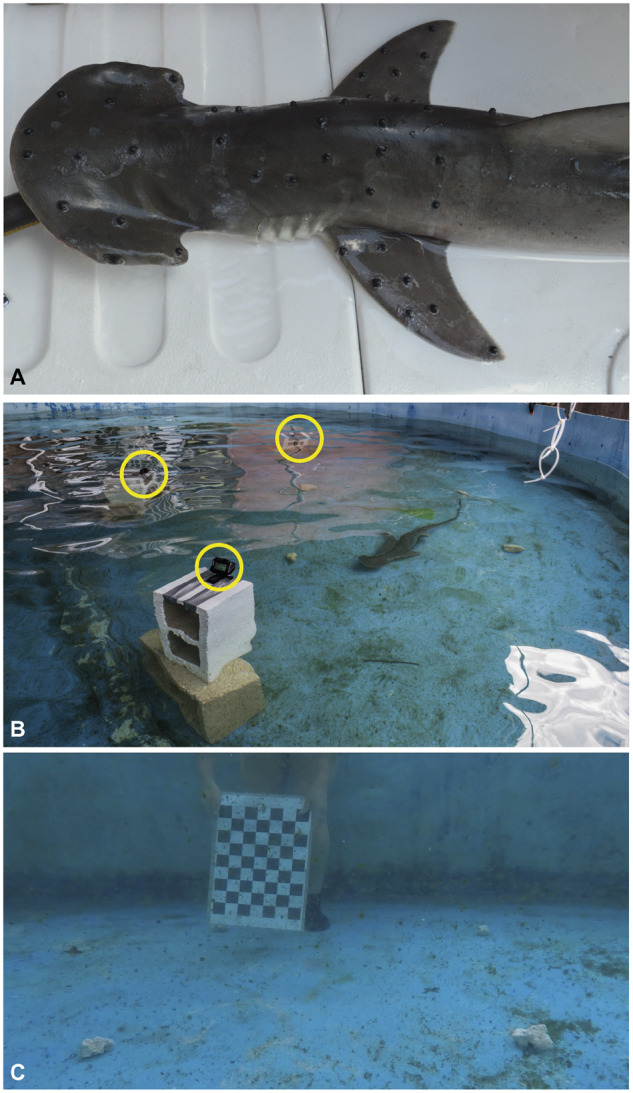
Camera set-up and bead placement for VROMM. (**A**) Black hemispherical beads were placed along the body and pectoral fins of four bonnethead sharks. (**B**) Three GoPro Hero 5 Black cameras (outlined in yellow) were mounted to cement blocks and angled at a common volume of interest, marked with white coral fragments. (**C**) The trial arena was calibrated for 3D analyses by taking images of a 7 square×9 square checkerboard at various regions throughout the volume.

Individuals were anesthetized via submersion in a 0.133 g/L MS-222 solution buffered with NaOH. Once ventilatory gill movement slowed to indicate anesthesia was in effect, individuals were placed on a surgical platform and intubated with fresh flow-through seawater. Black, hemispherical beads (5 mm diameter) were affixed to the trunk and pectoral fins with VetBond (3M Company, St. Paul, MN). At least five beads were positioned along the rigid anterior trunk and the proximal fin base near the leading edge (Fig. 1A). Bead placement lasted less than 3 min, and then the individual was returned to the semicircular 6 m diameter filming arena for a recovery period of approximately 1 h until normal ventilation and swimming behavior resumed.

### Volitional swimming trials

Methods described here were modified from Hoffmann et al. (2019). Three GoPro Hero 5 Black cameras were mounted to cement blocks and positioned along the lateral edge of the filming arena (GoPro, Inc., San Mateo, CA; Fig. 1B). Cameras were time synchronized using a flash of light and filmed at 1080 p ×1920 p, 60 fps, and we used a linear field of view, which removes the effect of fish-eye barrel distortion. A 31.5 cm×40.5 cm checkerboard (7 squares×9 squares) was used to calibrate the cameras for 3D analyses (Fig. 1C). Individuals were enticed to maneuver through the calibrated space by placing 2 cm diameter cylindrical pole in their path (Domenici et al. 2004). As individuals approached the side of the tank, the pole was slowly placed in front of the animal to avoid a startle response but still elicit a turn. Trials were chosen in which a clear yaw turn with minimal pitch adjustment occurred. For three individuals, three trials each met these criteria (*n* = 9), but for the fourth individual, only two trials were acceptable due to variable conditions within this flow through seawater system. In this study, we analyzed movement from only the inside fin during a turn because the body occluded the outside fin in the video reconstructions.

### Muscle stimulation trials

Upon completion of volitional swimming trials, individuals were euthanized via submersion in a 2 g/L MS-222 solution buffered with NaOH. Post mortem, individuals were fully submerged and suspended in a 190 L tank. Bipolar leads made from 57 μm diameter insulated alloy wire were placed in three pectoral fin muscles hypothesized to control maneuvering (dorsal pterygoideus [DP], ventral pterygoideus [VP], and cranial pterygoideus [CP]; Fig. 8). A 10 V, 30 Hz square wave pulse was applied to the targeted muscles one at a time via BK Precision 4052 signal generator (BK Precision Corporation, Yorba Linda, CA) to stimulate contraction. Stimulation lasted no longer than 2 s per muscle and we ensured that the fin returned to a resting position between trials. Muscle stimulation experiments were recorded with two GoPro Hero 5 Black cameras positioned approximately 45° to one another and focused on the tank with overlapping fields of view. Cameras were time synchronized with a flashing light and calibrated for 3D analysis using a checkerboard calibration object (Knörlein et al. 2016; Hoffmann et al. 2019).

Following muscle stimulation trials, the fin and pectoral girdle were dissected to confirm lead placement, and describe the muscle arrangement and articulations between the fin and girdle. Fin skeletal and muscle morphology differs among species and there are limited data on pectoral fin anatomy specific to the bonnethead (Wilga and Lauder 2000, 2001; Maia et al. 2012; Da Silva and De Carvalho 2015).

### 3D marker tracking

**Fig. 2:**
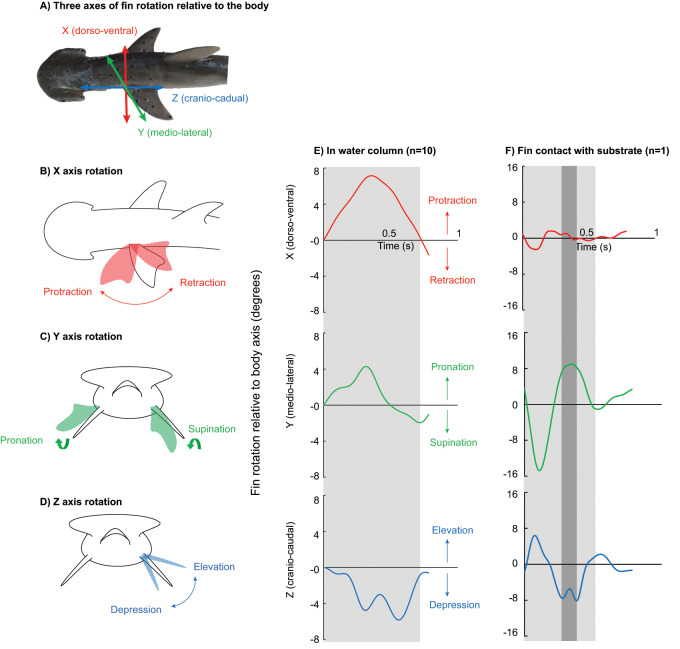
Fin rotation relative to the body axes in two sample trials demonstrating two turning strategies employed by the bonnethead shark. (**A**) JCS placement at the proximal fin base denotes three axes of fin rotation relative to the body. Rotation about the dorso-ventral body axis (*X*; red) represents (**B**) fin protraction and retraction, medio-lateral body axis (*Y*; green) represents (**C**) fin pronation and supination, and cranio-caudal body axis (*Z*; blue) represents (**D**) fin elevation and depression. (**E**) An exemplar trial demonstrating the pattern of fin rotation during turning in the water column, where the turning period is outlined in the light gray box. (**F**) Using this turning strategy fin rotation is more complex, where the fin is first retracted, supinated, and elevated, and then the fin is pronated and depressed to contact the substrate (darker gray box) before it returns to neutral.

For both volitional swimming and muscle stimulation trials, markers along the fin and body were tracked in 3D using XMALab v. 1.5.1 (Knörlein et al. 2016). Rigid bodies were created from markers on the leading edge of the pectoral fin and anterior trunk of the body to quantify the fin rotation relative to the body. Movement of the fin and trunk as rigid bodies was calculated in XMALab using five or more markers distributed in a constellation pattern to describe their relative motion. Rigid body transformations were applied to polygons that served as estimations of the fin and body in Autodesk Maya 2017 (San Rafael, CA). A joint coordinate system (JCS) was assigned to the proximal insertion at the base of the pectoral fin at the body axis (Fig. 2A;Camp and Brainerd 2015; Hoffmann et al. 2019). The Euler angle rotation (*α*; deg) was calculated for each of the three axes of rotation. Because individual Euler angle rotations were small (<25°) and were zeroed at the equator, we report total fin rotation as the scalar sum of rotation in all three axes (*β*; deg; Hoffmann et al. 2019).

### 2D whole body kinematics

Whole body kinematics were quantified using the *X*, *Z* coordinates representing the dorsal plane. Instantaneous linear velocity of the body (*V*; cm s^−1^) was calculated as the change in distance of a point at the first dorsal fin insertion over time, which was standardized by fork length (FL; cm) to derive swimming speed (*U*; body lengths s^−1^). The change in velocity of the body was calculated as the instantaneous velocity at the beginning of the turn minus instantaneous velocity at the frame of maximum total pectoral fin rotation (Δ*V*; cm s^−1^; Δ*U*, body lengths s^−1^). Instantaneous linear velocity and speed were also calculated at the dorsal tip of the caudal fin (*V*_cf_; cm s^−1^; *U*_cf_; body lengths s^−1^). All instantaneous velocity data were filtered with a low pass five point running average.

Instantaneous turning angle (*γ*) was calculated as the angular displacement of the dorsal fin insertion from the previous time step. Instantaneous angular velocity (*w*; Deg · s^−1^) was calculated as the instantaneous turning angle (*γ*) by the change in time. We calculated instantaneous turning radius of the dorsal fin insertion (*r*; cm) using the instantaneous turning angle (*α*) and the two adjoining segments in time (Porter et al. 2011). Turning radius is typically measured from the center of rotation, which we were unable to determine in this study; however, the dorsal fin insertion was visible throughout all trial and occurs approximately at the middle of the pectoral fins, which approximated the center of rotation in leopard sharks (Porter et al. 2011). The minimum turning radius was considered maximal turning performance.

A second set of turning metrics was calculated to assess performance of the overall turn. Turning angle (*θ*; deg) was calculated as the angle between the initial (*H*_i_) and final (*H*_f_) heading, where *H* is the hypotenuse between the two:

(1) $$\cos\left(\beta \right)=\;\frac{{H_{i}}^{2}+{H_{f}}^{2}-H^{2}}{2H_{i}H_{f}}.$$

Turning angular velocity (*w*_t_; Deg · s^−1^) was calculated as the change in angle over time:

(2) $$w_{t}=\;\frac{\theta }{t}.$$

Two metrics of body curvature were calculated to assess the role of axial bending in turning. Body curvature is represented by the bending coefficient (BC_1_) calculated as:

(3) $${\mathrm{BC}}_{1}=1-\;\frac{L}{\mathrm{TL}},$$

where L is the minimum distance between the head and the caudal peduncle during the turn (cm) and TL is the total length of the individual from the tip of the snout to a perpendicular line form the natural position of the caudal fin to the horizontal body axis (cm) (Brainerd and Patek 1998; Azizi and Landberg 2002; Kajiura et al. 2003; Porter et al. 2011). Body curvature was also assessed along the length of the whole body as Porter et al. (2009) demonstrate that BC_1_ may overestimate body curvature due to flexibility of the tail so we calculated a second body curvature metric as

(4) $${\mathrm{BC}}_{2}=1-\;\frac{L}{\mathrm{FL}},$$

where FL is the length of the individual from the tip of the snout to the fork of the caudal fin (cm).

### Data analysis

We report the fin rotation angle about each axis (*α*) from the frame of maximum total rotation (*β*) as a range and the mean ± standard error of the mean. The magnitude of rotation in each axis was compared using a one-way ANOVA. The effect of body and fin movement on turning performance was examined using simple linear regressions between body curvature measurements, and fin rotation, turning angle (*θ*), and turning angular velocity (*w*_t_). To determine the effect of whole body kinematics on turning performance, we applied a generalized linear model with forward stepwise selection to determine the best fit model. The four turning performance response variables were maximum instantaneous angular velocity (*w*), angular velocity of the whole turn (*w*_t_), minimum instantaneous turning radius (*r*), and instantaneous turning radius standardized by TL (*r*_st_). Predictor variables were pectoral fin rotation in all three axes (*α_x_*, *α_y_*, and *α_z_*), total fin rotation (*β*), instantaneous linear velocity at the frame of maximum total pectoral fin rotation (*V*), instantaneous linear speed at the frame of maximum total pectoral fin rotation (*U*), change in velocity (Δ*V*) and swimming speed (Δ*U*), average caudal fin velocity (*V*_cf_) and speed (*U*_cf_), bending coefficients (BC_1_ and BC_2_), and individual. Our sample size of trials (*n* = 11) was limited due to the challenges associated with keeping bonnethead sharks in captivity and the limited amount of time to conduct turning trials post-bead placement anesthesia, and we acknowledge the limitations. Future studies should examine a larger sample size and other species to strengthen relationships between turning performance and fin and body movements.

Fin rotation and turning performance variables for the bonnethead were compared with the same variables measured for Pacific spiny dogfish in a previous study (Hoffmann et al. 2019). Fin rotation about each axis, total fin rotation, change in velocity, turning angle, and turning angular velocity were compared between species in a one-way ANOVA.

## Results

### Pectoral fin kinematics

In all turning trials analyzed, the pectoral fin rotated about all three body axes (Fig. 2). For 10 of the 11 trials, the inside fin was protracted, pronated, and depressed (Fig. 2E). In one trial, the individual first retracted, supinated, and elevated the fin, quickly followed by fin pronation and depression to contact the substrate during turning (Fig. 2F). We removed this trial from further analyses as it represents an alternative maneuvering strategy.

**Fig. 3:**
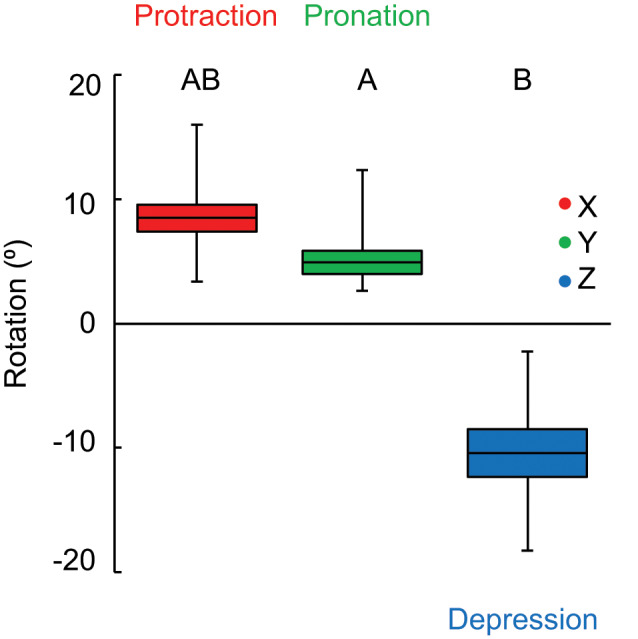
Range of pectoral fin rotation about the body axes during turns, in which the fin did not contact the substrate. The magnitude of rotation only differed between *Y* and *Z* axes, where *X* axis rotation was the intermediate. Boxes represent the mean (middle line) ± the standard error of the mean, and whiskers represent the minimum and maximum values. During turning, the fin was protracted (*X*), pronated (*Y*), and depressed (*Z*). Significant differences are denoted by letter.

We observed positive *X* axis rotation, representing fin protraction, ranging from 3° to 16° (Figs. 2B and 3; 8.5° ± 1.1° SE). *Y* axis rotation was also positive, indicating fin supination, ranging from 3° to 12° (Figs. 2C and 3; 5.0° ± 0.9°). Rotation about the *Z* axis was negative, representing fin depression, ranging from −18° to −2° (Figs. 2D and 3; 10.4° ± 1.9°). The magnitude of rotation did not differ between *X* and *Z* or *X* and *Y* axes, but *Z* axis rotation was significantly greater than *Y* (Fig. 3; *F*_2,29_ = 4.2283, *P* = 0.0253). Total fin rotation ranged from 15° to 35° (24° ± 2.3°).

Point tracking precision was calculated as the standard deviation of the intermarker distance within a rigid body (Knörlein et al. 2016). In this study, we assume that the fin base and body are rigid, despite lacking true rigid elements (i.e., bones). Marker based XROMM studies report mean SD of intermarker distance <0.1 mm, and a previous video reconstruction of moving morphology (VROMM) study on Pacific spiny dogfish reports the mean SD of intermarker distance <0.7 mm, which is <0.02% of the animal’s TL (Hoffmann et al. 2019). In this study, there was no difference in the SD of intermarker distance between rigid bodies (fin base vs. body) or among individuals. Mean point tracking precision for all trials and rigid bodies was 2.19 mm. We hypothesize that the increase in precision error observed here compared with the previous shark VROMM study is the result of a 4× increase in the volume of interest and larger study organisms (Hoffmann et al. 2019). Even so, precision error in this study is still <0.3% of the bonnethead shark’s total body length.

### Turning performance

**Fig. 4:**
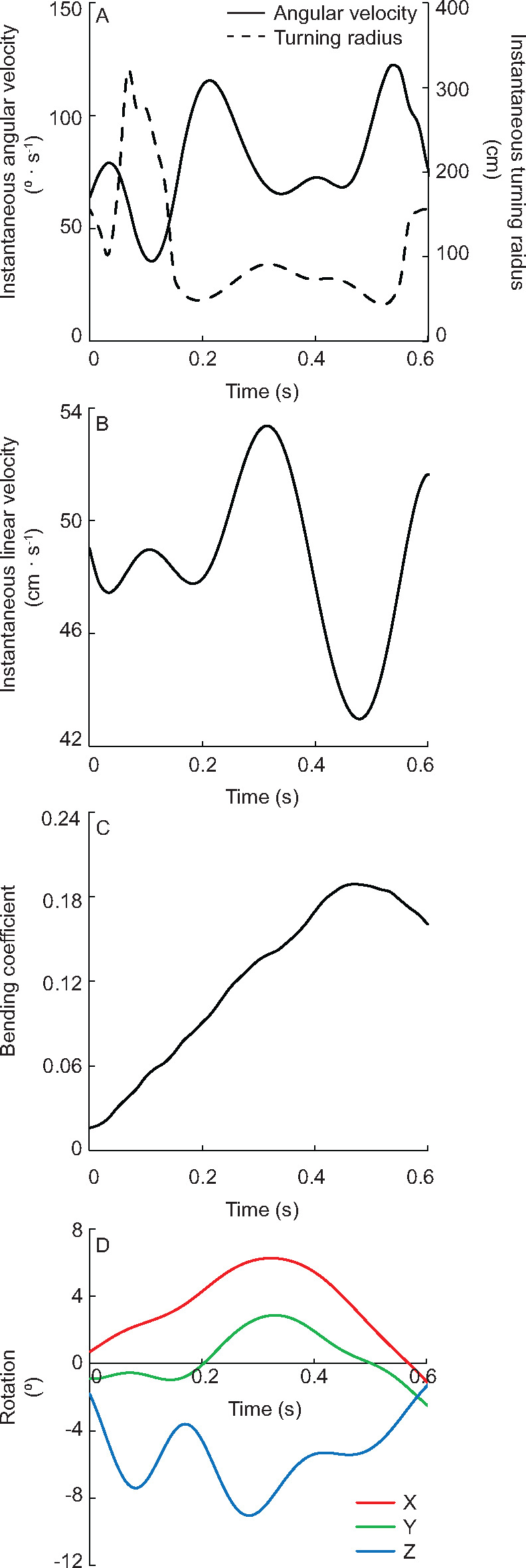
Sample whole body kinematics and turning performance. **A**) During turning, angular velocity increases while turning radius decreases. **B**) Simultaneously, instantaneous linear velocity decreased for all trials. **C**) Bending coefficient and (**D**) fin rotation both increase during the turn.

During turning, we observed changes in instantaneous angular velocity, instantaneous linear velocity, instantaneous bending coefficient, and instantaneous pectoral fin rotation that occurred at similar time points (Fig. 4). To better assess the overall performance of the turn, we report relationships between overall turning angular velocity (*w*_t_), minimum turning radius (*r*), maximum bending coefficient (BC_1_ and BC_2_), and average caudal fin velocity throughout the duration of the trial. To standardize among all trials, we analyzed instantaneous linear velocity and fin rotation about each axis from the same frame of maximum total fin rotation since these variables were most closely related in timing and represented the midpoint, or “peak,” of the turn.

As with pectoral fin rotation values, we observed a range of body bending and velocities among trials. Both body bending coefficients were variable, ranging from BC_1_ = 0.19–0.53 (0.37 ± 0.03) and BC_2_ = 0.10–0.48 (0.26 ± 0.04). Instantaneous linear velocity from the frame of maximum total rotation approximately doubled between the fastest (63.94 cm s^−1^, 1.02 body lengths s^−1^) and slowest (37.52 cm s^−1^, 0.60 body lengths s^−1^) trials (51.22 ± 2.44 cm s^−1^; 0.76 ± 0.03 body lengths s^−1^). Δ*V* varied even more, more than tripling from the greatest (26.61 cm s^−1^, 0.38 body lengths s^−1^) to the least (7.61 cm s^−1^, 0.12 body lengths s^−1^) difference (15.90 ± 2.0 cm s^−1^, 0.23 ± 0.03 body lengths s^−1^). Of the velocity variables calculated, average caudal fin velocity was the least variable, ranging from 66.01 to 92.57 cm s^−1^ and 1.00 to 1.34 body lengths s^−1^ (77.42 ± 2.26 cm s^−1^; 1.16 ± 0.03 body lengths s^−1^).

**Fig. 5:**
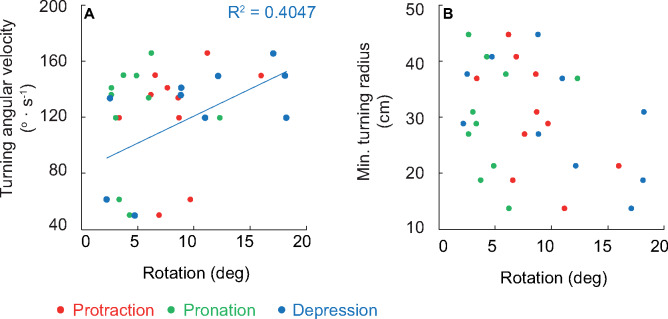
Fin rotation relative to turning performance. (**A**) Fin depression was the only axis of rotation significantly related to turning angular velocity. (**B**) Fin rotation in separate axes was not related to turning radius. Significance (*P* < 0.05) is denoted by the corresponding trendline and *R*^2^ value.

**Fig. 6:**
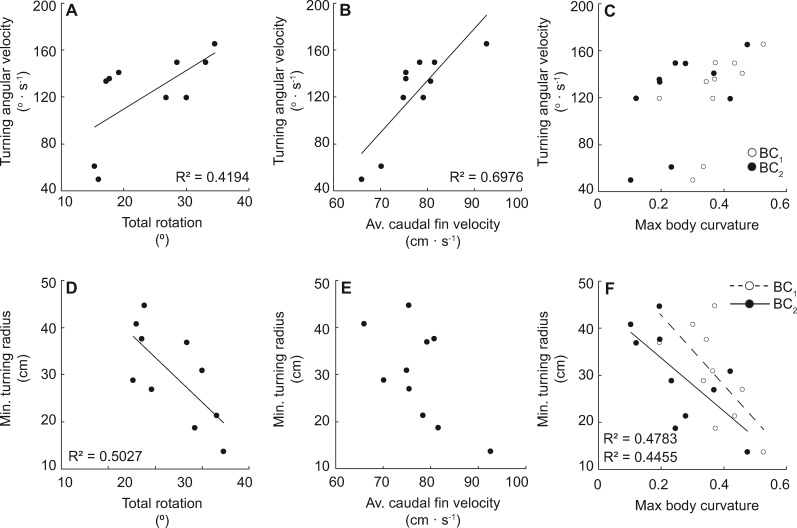
Whole body kinematics relative to turning performance. Total fin rotation (**A**) and average caudal fin velocity (**B**) were positively related to turning angular velocity, while neither of the BC metrics were (**C**). Total fin rotation (**D**) and both BC metrics (**F**) were negatively related to minimum turning radius, while average caudal fin velocity was not (F). Significance (*P* < 0.05) is denoted by the corresponding trendline and *R*^2^ value.

When separated by axes of rotation, only fin depression was significantly related to the angular velocity of the turn, and none of the axes of fin rotation were related to minimum turning radius (Fig. 5; *R*^2^ = 0.4047, *P* = 0.0480). The interaction term between *X*, *Y*, and *Z* rotation was significantly related to minimum turning radius but not angular velocity, which we hypothesize is due to two slow outlier trials where *w*_t_ was <60 deg ·  s^−1^ (*r*_st_: *F* = 10.0633, *P* = 0.0131; *w*_t_: *F* = 0.2694, *P* = 0.6178). Total fin rotation (Fig. 6A;*R*^2^ = 0.4191, *P* = 0.0429) and average caudal fin velocity (Fig. 6B;*R*^2^ = 0.6976, *P* = 0.0026) were positively related to turning angular velocity while neither of the body curvature metrics were (Fig. 6C). Total fin rotation was also related to minimum turning radius (Fig. 6D;*R*^2^ = 0.5027, *P* = 0.0217), as were both metrics of body curvature (Fig. 6F; BC_1_: *R*^2^ = 0.4783, *P* = 0.0267; BC_2_: *R*^2^ = 0.4455, *P* = 0.0350), while average caudal fin velocity was not (Fig. 6E).

We ran two sets of models to predict turning performance, one with raw velocities and another in which velocities were standardized by body length. For predicting overall angular velocity, total fin rotation and BC_1_ were significant factors for both models, but only the model including Δ*V* and average caudal fin velocity was significant (Table 1). Further, this model explained 92% of the variation in our overall angular velocity data. Both sets of models to predict turning radius and standardized turning radius were significant included BC_1_ and individual, accounting for up to 90% of the variation in the data (Table 1). For all of the models that included raw velocity metrics, Δ*V* was a significant factor. None of the models that considered standardized velocity metrics included those variables in the best fit model. In all but one model, individual was a significant factor and is likely driving the kinematic response. We hypothesize that this is likely related to the limited sample size of individuals (*n* = 4) and trials analyzed per individual (*n* = 3).

**Table 1:** Best fit models for turning performance metrics.

Turning performance	*R* ^2^	Adj. *R*^2^	*P* < 0.05	*F*	Parameters			
*w*_t_ (deg · s^−1^)	0.9213	0.8584	0.0057	14.6367	Δ*V*	*β*	Av. _*V*cf_	BC_1_
*w* (deg · s^−1^)	0.9166	0.7497	0.0952	5.4938	Δ*V*	*V*	BC_2_	Individual
*r* (cm)	0.8871	0.8307	0.003	15.7151	Δ*V*	BC_1_	Individual	
*r*_st_ (body lengths)	0.9094	0.837	0.0081	12.5538	Δ*V*	BC_1_	BC_2_ individual	
**Turning performance: size standardized**								
*w*_t_ (deg · s^−1^)	0.9942	0.9481	0.1652	21.5528	*β* BC_1_	BC_2_	Individual	
*w* (deg · s^−1^)	0.8776	0.6329	0.161	3.5857	*β*	BC_1_	BC_2_	Individual
*r* (cm)	0.9046	0.8283	0.0092	11.8562	BC_1_	Individual		
*r*_st_ (body lengths)	0.9199	0.8557	0.006	14.3460	BC_1_	Individual		

### Muscle stimulation

**Fig. 7:**
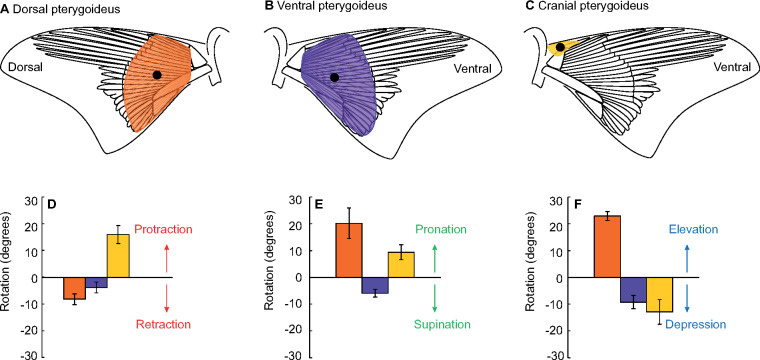
Fin rotation relative to the body from stimulation of three target muscles in the pectoral fin. DP (**A**; orange), VP (**B**; purple), and CP (**C**; yellow). Lead placement targeted the middle of the muscle body, represented by black dots. (**D**–**F**): Fin rotation relative to the *X* (D), *Y* (E), and *Z* (F) axes described in Fig. 2. The CP was the only muscle to produce fin protraction (D) whereas the DP and VP both resulted in retraction. The DP and CP both pronated the fin (E) and the VP supinated the fin. The DP was the only fin elevator (F) and stimulation of the VP and CP both resulted in fin depression. Error bars represent standard error of the mean.

Stimulation of each targeted muscle resulted in fin rotation about all three body axes. Negative *X* axis rotation (Fig. 2B: fin retraction) occurred with stimulation of the DP (Fig. 7D; −8.2° ± 2.1°) and the VP (Fig. 7D; −3.9° ± 2.0°). The CP was the only muscle to protract the fin (*X* axis; Fig. 7D; 15.9° ± 3.4°). The DP and CP pronated the fin (*Y* axis; Fig. 7E; 20.2° ± 5.7°, 9.4° ± 2.7°, respectively), while the VP was the only fin supinator (Fig. 2C;*Y* axis; −5.9° ± 1.5°). Finally, the DP was the only muscle to produce fin elevation (Fig. 2D:*Z* axis; Fig. 7F; 22.9° ± 1.7°). Both the VP and CP acted as fin depressors (Fig. 7F; −9.3° ± 2.5°, −12.9° ± 4.6°).

Fin rotation and turning performance values for the bonnethead were compared with previous data on the Pacific spiny dogfish (Hoffmann et al. 2019). The polarity of *Y* axis rotation differed between the bonnethead (pronation) and Pacific spiny dogfish (supination), but the absolute value of rotation did not differ. All other kinematic variables were similar between the two species.

## Discussion

### Pectoral fin kinematics

For 10 of 11 trials, we observed that the inside pectoral fin was protracted, pronated, and depressed during yaw turning in the water column (Figs. 2B–D, 3, and 4). Fin depression changes the negative dihedral angle of the fin to the body, which is previously shown to increase maneuverability (Wilga and Lauder 1999, 2000). Additionally, pectoral fin rotation is a significant factor in three of the models that predict turning angular velocity (*w*, *w*_t_; Table 1), demonstrating that fin rotation in the bonnethead contributes to turning performance. In this study, we describe pectoral rotation relative to the body axes with the caveat that the fin may also be undergoing conformational changes, which are not captured in these data (Wilga and Lauder 2000, 2001). Although we were unable to capture the motions of the fin outside to body curvature in this study, previous studies note significant differences in the fin area presented from a dorsal view during yaw turning in the bonnethead (Kajiura et al. 2003). In this study, we were able to quantify the magnitude and describe the polarity of inside fin rotation in three axes and confirm that the inside fin is actively rotated during routine yaw maneuvering.

Of the three axes in which the pectoral fin rotates, only *Z* axis rotation (fin depression) was related to turning performance metrics, which was the same for the Pacific spiny dogfish (*w*_t_; Hoffmann et al. 2019; Fig. 5A). For Pacific spiny dogfish, pectoral fin depression was also the axis of greatest fin rotation, though in the bonnethead *Z* and *X* and *Y* and *X* axis rotation were not different (Hoffmann et al. 2019; Fig. 3). Additionally, no axis of rotation was related to turning radius (Fig. 5B). For both species, these results suggest that the combination of fin rotation is more significant to turning performance than any one axis considered alone.

The primary result of fin pronation is a change in angle of attack, or orientation to flow. Assuming that pectoral fin angle of attack, as previously described in the literature, is largely a factor of long axis rotation (*y* axis), we report a similar range of fin pronation found in some species (leopard, sandbar, sand tiger, spiny dogfish, and white sturgeon; Fig. 3; Wilga and Lauder 1999, 2000; Fish and Shannahan 2000). During vertical maneuvering, sturgeon and leopard shark pectoral fins are rotated synchronously generating thrust to reorient the anterior body to rise (Wilga and Lauder 2000). Asynchronous fin depression and pronation would destabilize the body, thereby increasing maneuverability (Fish 2002; Fish et al. 2006; Goldbogen et al. 2013; Segre et al. 2016). Indeed, we document that total fin rotation is related to both turning angular velocity and minimum turning radius (Fig. 6A, D), and was a significant model factor in predicting turning angular velocity (Table 1).

### Whole body kinematics

Body bending is a major factor in shark turning performance (Kajiura et al. 2003; Domenici et al. 2004; Porter et al. 2009, 2011). Both metrics of bending coefficient (BC_1_ and BC_2_) are related to minimum turning radius (Fig. 6F). The BC_1_ we calculated for routine bonnethead turning are substantially smaller than those reported for other shark species (Table 2). Many previous studies focus on maximal turning performance, rather than routine maneuvering, despite observations that most maneuvering is more moderate (Webb 1991; Wu et al. 2007a). We hypothesize that the difference in BC we report here is a factor of the moderate turning behaviors we observed.

**Table 2:** Turning performance metrics as they compare to previous studies. All values represent the means as reported or were adapted from figures.

Species	**Turning velocity (**deg · s^−1^**)**	Turning radius (%TL)	BC_1_	BC_2_	Citation
Scalloped hammerhead	470	18.3	0.64	–	Kajiura et al. (2003)
Sandbar	250	19.3	0.57	–	Kajiura et al. (2003)
Bonnethead (electrical stimulus)	–	–	0.59	–	Kajiura et al. (2003)
Horn shark	–	–	0.65	0.6	Porter et al. (2009)
Brownbanded bamboo shark	–	–	0.8	0.7	Porter et al. (2009)
Whitespotted bamboo shark	–	–	0.75	0.7	Porter et al. (2009)
Epaulette shark	–	–	0.9	0.75	Porter et al. (2009)
Leopard shark	187	0.6	0.75	0.7	Porter et al. (2009, 2011)
Spiny dogfish (slow escape)	706	7.4	0.45		Domenici et al. (2004)
Spiny dogfish (fast escape)	1221	6	–	–	Domenici et al. (2004)
Pacific spiny dogfish (routine turn)	27.4	–	–	–	Hoffmann et al. (2019)
Bonnethead (routine turn)	150.4	37.4	0.37	0.26	Present study
Undulatory rays	20.1	2.2	–	–	Parson et al. (2011)
Oscillatory rays	24.8	2.1	–	–	Parson et al. (2011)
Manta ray	18.26	38	–	–	Fish et al. (2018)
Reef fish	730	6–9	–	–	Gerstner (1999)
Koi carp	100–1000	31.5	–	–	Wu et al. (2007b)
Angelfish	–	6.5	–	–	Domenici and Blake (1991)
Smallmouth bass	–	13	–	–	Webb (1983)
Dolphin fish	–	13	–	–	Webb and Keyes (1981)
Yellowtail	–	23	–	–	Webb and Keyes (1981)
Rainbow trout	–	18	–	–	Webb (1976)
Yellowfin tuna	–	47	–	–	Blake et al. (1995)
Spotted boxfish	107–218	3.25	–	–	Walker (2000)
Painted turtle	136.4	24.77	–	–	Rivera et al. (2006)
Bottlenose dolphin	430.6	21.5	–	–	Maresh et al. (2004)
Male sea lion	513.8	11	–	–	Fish et al. (2003)
Female sea lion	599.2	19	–	–	Fish et al. (2003)
Brief squid (feeding)	288.3–302.6	30–60	–	–	Jastrebsky et al. (2017)
Brief squid	110.3	0.36	–	–	Jastrebsky et al. (2016)
Dwarf cuttlefish	54.8	4.0	–	–	Jastrebsky et al. (2016)
Whirligig beetle	1790.2	86	–	–	Fish and Nicastro (2003)

### Turning performance

The values we document for turning angle and turning angular velocity are comparable to Pacific spiny dogfish, but are substantially less than those previously reported for other shark species (Table 2). Previous studies document yaw maneuvering in the context of prey locating or escape responses, and we hypothesize that the turning behavior captured in our data represents slow, steady maneuvering rather than reacting to stimuli (Table 2). The turning rate and radius documented here are most comparable to leopard shark turning, but the BC quantified in those turns were much greater since maximal turning performance was targeted in that instance (Table 2). Koi carp had similar turning radii during routine maneuvering behavior, though turning rate was much greater than we found in the bonnethead (Wu et al. 2007b; Table 2). Routine turning in the manta ray also had a comparable turning radius to the bonnethead but at a much slower rate (Fish et al. 2018; Table 2). The manta ray and yellowfin tuna both had larger turning radii than we found for routine turns in the bonnethead, which is likely a factor of differences in body stiffness. Understanding the interactions among morphology, ecology, and context (e.g., routine maneuvering, foraging, predator response, etc.) is critical to analyzing the function of different animals among their ranges of behaviors and environments.

Turning angular velocity (*w*_t_) and minimum turning radius (*r*) were related to a number of whole body kinematic variables (Fig. 6). Similar to the relationships previously described for the Pacific spiny dogfish, *Z* axis and total fin rotation are both significantly related to turning angular velocity (Hoffmann et al. 2019; Figs. 5A and 6A). We found a greater than three-fold increase between the fastest (*w_t_* = 165.4 deg · s^−1^) and slowest (*w_t_* = 49.9 deg · s^−1^) trials, and total fin rotation doubled and fin depression more than tripled with angular velocity. Total fin rotation is also significantly related to minimum turning radius (Fig. 6D). Together, these data suggest that increasing fin rotation plays a role in creating tighter, faster turns in the bonnethead shark.

When considering all of the predictor variables together, we found that Δ*V* and BC_1_ were consistently significant factors in the models that were not size corrected (Table 1). The best fit model for angular velocity (*w*_t_) explains 92% of the variation in the data and included Δ*V*, total fin rotation, average caudal fin velocity, and BC_1_. For both sets of models, body curvature appears to be the largest factor in predicting turning radius, and in combination with other factors explain >88% of the variation in the data. Considering the high *R*^2^ values observed in both sets of models, we hypothesize that the kinematic variables considered in this study (pectoral fin movements, body curvature, linear velocity near the center of mass, and caudal fin velocity) capture a large portion of the maneuvering effort. Other kinematic factors known to contribute to swimming performance include changes in pectoral fin conformation, caudal fin displacement and stiffness, and dorsal fin movements, which may account for the variation not explained in the present study (Wilga and Lauder 2000, 2001; Flammang 2010; Maia et al. 2012; Maia and Wilga 2013, 2016).

### Muscle stimulation

Bonnethead sharks rotate their inside fin relative to all three body axes and post-mortem stimulation confirmed functional hypotheses of the associated pectoral fin muscles. Three muscles are directly affiliated with the fin itself and were previously shown to play a role in actuation: the DP, VP, and CP (Fig. 7; Maia et al. 2012; Hoffmann et al. 2019). Our experiments showed that stimulation of each individual muscle resulted in fin rotation about all three axes (Fig. 7).

The DP originates on the scapulo-coracoid and axial musculature posterior to the pectoral girdle and fans out distally over the three basal cartilages to insert on the intermediate radials (Fig. 7). Stimulation of the DP resulted in fin retraction, pronation, and elevation (Fig. 7). Fin elevation is previously attributed to DP activity, and this pattern of rotation relative to the body axes is the same as the Pacific spiny dogfish (Marinelli and Strenger 1959; Maia et al. 2012; Hoffmann et al. 2019). The VP also produced the similar rotation patterns in the bonnethead and the Pacific spiny dogfish: retraction, supination, and depression (Fig. 7; Hoffmann et al. 2019). On the ventral side of the fin, the VP also originates on the scapulo-coracoid and the axial musculature, and it inserts on the intermediate radials (Fig. 7). In two axes (*Y* and *Z*), the DP and VP are antagonistic muscles: the DP pronates and elevates the fin while the VP supinates and depresses the fin (Fig. 7). Both muscles retracted the fin, likely resulting from the muscle fibers fanning out distally at an oblique angle to the body axis (Fig. 7).

The only muscle to produce a different pattern of rotation in the bonnethead compared with the Pacific spiny dogfish was the CP. In the bonnethead, the CP originates antero-medially to the scapulo-coracoid (Fig. 7). Unlike squalids where the CP has insertions on both sides of the fin, the bonnethead CP is localized to the anterior margin of the scapulo-coracoid and does not fan out distally into the fin (Marinelli and Strenger 1959; Hoffmann et al. 2019). Stimulation of the CP protracted, pronated, and depressed the fin (Fig. 7C–F). In the Pacific spiny dogfish, the CP supinated the fin, and varied EMG lead placement resulted in both depression and elevation of the fin (Hoffmann et al. 2019). *Y* axis rotation was the only difference in the pattern of fin rotation between the two species during turning, where bonnethead sharks pronated the fin and Pacific spiny dogfish supinated the fin (Fig. 7; Hoffmann et al. 2019). We hypothesize that the dissimilar muscle morphology and function between these two species represent differences in the pattern of fin rotation observed during volitional maneuvering. These results demonstrate the importance of evaluating species level differences in morphology before generalizing function among groups.

## Conclusion

In this study, we document significant relationships between turning performance metrics (angular velocity, turning radius) and a suite of kinematics variables (Fig. 6 and Table 1). We hypothesize that the decreased agility and maneuverability quantified in this study compared with other shark turning studies are due to the context of the behaviors analyzed. In this study, we described routine turning maneuvers and compare with maximal turning performance and/or stimulated responses in which individuals demonstrated increased agility and maneuverability. Bonnethead turning observed in this study was comparable to routine turning previously described for the Pacific spiny dogfish, routine turning in manta rays, and (to some degree), turning in leopard sharks. Despite our growing understanding of locomotion in aquatic vertebrates, the kinematics of routine maneuvering remains to be fully explored. Future studies should consider a suite of kinematic variables to capture a full understanding of routine maneuvering to better extrapolate this performance on a larger scale and to more natural behaviors, such as the large scale migrations that are common among shark species.
